# Treatment and Prognosis of Vogt–Koyanagi–Harada Disease: Real-Life Experience in Long-Term Follow-Up

**DOI:** 10.3390/jcm11133632

**Published:** 2022-06-23

**Authors:** Massimo Accorinti, Maria Carmela Saturno, Ludovico Iannetti, Priscilla Manni, Davide Mastromarino, Maria Pia Pirraglia

**Affiliations:** Ocular Immunovirology Service, Department of Sense Organs, Sapienza University of Rome, 00161 Rome, Italy; mariacarmela.saturno@virgilio.it (M.C.S.); ludovico.iannetti@gmail.com (L.I.); priscilla.manni@uniroma1.it (P.M.); mastromarino.d11@gmail.com (D.M.); mariapia.pirraglia@tiscali.it (M.P.P.)

**Keywords:** Vogt–Koyanagi–Harada disease, treatment, prognosis, uveitis, complications

## Abstract

Background: Vogt–Koyanagi–Harada (VKH) disease is a form of uveitis that is rare in Western countries. The aim of this study was to report on the long-term real-life treatment and prognosis of VKH in Italy. Methods: The clinical features, complications, and final visual acuity were retrospectively evaluated in 38 patients with VKH (mean follow-up: 120 months) globally, according to oral or intravenous corticosteroid treatment at onset and subsequent immunosuppressive therapy. Results: The mean final visual acuity was 0.13 ± 0.4 logMAR, which was a significant increase from the baseline (*p* < 0.0001). The patients who received intravenous rather than oral corticosteroids relapsed less (*p* = 0.026), with fewer relapses/patient/month of follow-up (*p* < 0.0001), and showed less frequent sunset glow fundus (33.3% versus 55%) and more relapse-free cases after induction therapy (*p* = 0.007). Delayed immunosuppressive therapy (median: 180 days from the onset of symptoms) reduced the rate of sunset glow fundus. The onset of sunset glow fundus was associated with a worse final visual acuity (*p* = 0.006). Conclusion: The long-term prognosis of VKH is quite good. Intravenous corticosteroids given at the onset of VKH are more effective than oral corticosteroids. Even if it is not given immediately after symptoms onset, immunosuppressive therapy is able to reduce the incidence of sunset glow fundus and to improve the final visual prognosis.

## 1. Introduction

Vogt–Koyanagi–Harada disease (VKH) is a granulomatous inflammatory disorder affecting the eyes, auditory system, meninges, and skin [1,2].

It begins with a prodromal stage, lasting a few days, which is characterized by a viral-like illness, frequently associated with headache, neck stiffness, and confusion, followed by an acute uveitis stage, marked by the appearance of bilateral diffuse choroiditis, papillitis, and exudative retinal detachment, with or without signs of inflammation in the anterior chamber. The progression of the disease takes place during the convalescent stage, with nummular chorioretinal depigmented scars and retinal pigment epithelium clumping and migration.

In some patients, who either are undertreated or do not adequately respond to therapy, a chronic recurrent stage may develop, with multiple uveitis recurrences, located either in the anterior or the posterior segment of the eye. Usually, during the convalescent or chronic recurrent stages, skin manifestations develop, namely vitiligo and poliosis [1,2,3].

In the initial stage, VKH usually responds to high-dose corticosteroid therapy, either administered orally or intravenously [1,2,4,5,6]. Early therapy can help to prevent the progression of the disease to the later stages of its natural course. Nevertheless, disease recurrence can happen in some patients at medium-to-long-term follow-up points, and the disease might progress to the onset of diffuse depigmentation of the eye (sunset glow fundus) [7,8,9]. Immunosuppressive therapy can be added to the treatment regimen for VKH in different stages of the disease to increase the potency of corticosteroid therapy, reduce the total dosage of corticosteroid therapy needed to control the disease, and when corticosteroids are contraindicated because of systemic underlying disease (e.g., diabetes) [1,2,10,11]. Recently, some authors proposed the use of immunosuppressive drugs in the very early stage of the disease [12,13] to reduce the number of relapses and prevent the onset of sunset glow fundus, which might be associated with reduced visual function, even if good visual acuity persists.

Therefore, it seems crucial to place VKH patients on high-dose corticosteroid therapy, and eventually immunosuppressive drugs, within 2–3 weeks of symptom onset, to limit the number of relapses, as well as the rate of onset of sunset-glow fundus [1,5,12,13].

This study aimed to provide additional data on the long-term real-life treatment and prognosis of VKH seen at a tertiary referral center located in an area with a relatively low incidence of this disease.

## 2. Materials and Methods

In this retrospective study, we included all patients who were subject to follow-up at the Ocular Immunovirology Service, Department of Sense Organs, Sapienza University of Rome, with a diagnosis of VKH and a follow-up longer than 12 months, at the time of the study. Additionally, all patients included in this study needed to have a detailed description of their clinical situation at the time of symptom onset, along with all data concerning the course of the disease, the occurrence of complications, and the therapy administered (drugs and dosages) from uveitis onset to our first examination.

The diagnosis of VKH was made according to the revised diagnostic criteria published in 2001 [14]. All patients fulfilled the criteria of acute VKH at the time of uveitis onset, with neurologic involvement (either meningismus, headache, scalp sensitivity, tinnitus or hearing loss). At the end of our follow-up, we categorized the form of the disease into complete, if the patients also experienced integumentary findings (poliosisis, vitiligo, alopecia), or incomplete, if no other systemic symptoms appeared.

All patients who attended our center needed to sign an informed consent form before being examined, for use in an anonymous form of their clinical data for research purposes. The study was conducted following the tenets of the Declaration of Helsinki and was approved by the ERB of our institution.

For each study participant, we considered the clinical features at presentation drawn from the patients’ personal files, the period from symptom onset to initial therapy and to our examination, therapy given at presentation (drugs and dosages), changes in best-corrected visual acuity (measured with a decimal chart and converted to the logarithm of the minimum angle or resolution equivalents for statistical analysis—logMAR), complications (cataracts, intraocular pressure (IOP) increase, neovascular membrane), the appearance of sunset glow fundus, and the number of patients out of therapy.

**Figure 1:**
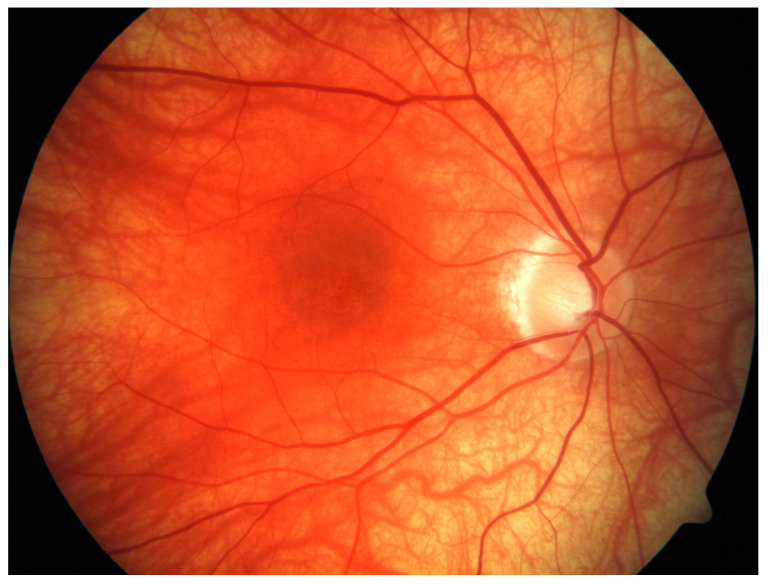
Typical sunset glow fundus of one patient included in the study: diffuse orange-red discoloration of the fundus with peripapillary atrophy and mottling of the pigment epithelium in the macular region.

IOP ≥ 24 mmHg detected in two separate observations was defined as IOP increase. Sunset glow fundus was defined as the observation of a diffuse orange-red discoloration of the fundus due to depigmentation (Figure 1).

According to the type of therapy received at the time of uveitis onset, the patients were divided into 2 groups: group 1 included patients who received oral corticosteroids (1 mg/kg/day of prednisone as induction therapy, which was slowly reduced), while group 2 included those who received intravenous methylprednisolone (1000 mg for 3 to 5 days, followed by oral prednisone, which was slowly reduced). Furthermore, study participants were also investigated regarding their use of immunosuppressive drugs at any time during the follow-up and the treatment received at symptom onset (i.e., either intravenous or oral corticosteroids).

Oral azathioprine (2–3 mg/kg/day), subcutaneous methotrexate (20 mg/week), oral cyclosporine A (5 mg/kg/day), and subcutaneous adalimumab (40 mg twice monthly) were immunosuppressive drugs used during the follow-up.

Four patients received immunosuppressive drugs before our examination (one cyclosporine A and methotrexate subsequently; one cyclosporine A alone; two methotrexate) and we administered azathioprine to the other fifteen. Eight of these were treated as soon as possible after our examination, and seven during follow-up. Two out of these seven refused immunosuppressive therapy at the time of our first examination; the other five preferred to delay the immunosuppressive therapy in case of uveitis relapse or corticosteroid-related adverse events.

After its availability, adalimumab was used in three patients, with two being unresponsive to azathioprine and one to cyclosporine, azathioprine, and methotrexate.

In cases of uveitis relapse, in some patients with asymmetrical involvement and in those who could not use or refused systemic corticosteroid therapy, we administered either orbital floor periocular injections (methylprednisolone or triamcinolone acetonide 40 mg), or intravitreal implantation of dexamethasone 700 μg.

At the time of our observations and during follow-up, all the patients were submitted to fluorescein angiography (FA), indocyanine green angiography (ICG), optical coherence tomography (OCT), and enhanced-depth imaging optical coherence tomography (EDI-OCT) according to their availability and to the clinical course of the disease.

The main outcome measures studied in these groups included the period from symptom onset to initial therapy and to our examination, changes in best-corrected visual acuity, clinical features at presentation, number of relapses and relapses per patient per month of follow-up rate, the total dosage of systemic corticosteroid therapy, the number of periocular injections, the number of dexamethasone intravitreal implants, and the presence of complications, as previously indicated.

In this study, any change in the amount of ocular inflammation in the anterior chamber (cells and flare), vitreous changes (vitreous haze and vitreous cells), retina and choroid (onset of new foci of choroiditis, optic disc hyperemia/oedema, focal areas of subretinal fluids or bullous serous retinal detachment) was considered as a relapse. These are stricter criteria than those proposed by the Standardization of Uveitis Nomenclature Study Group (SUN) for uveitis [15]; however, the criteria were chosen to ensure we did not underestimate the number of recurrences. Therefore, patients were classified as having a relapse of uveitis when any presence of or increase in inflammation was detected in the anterior or posterior segments of the eye.

All data from categorical variables are presented as frequencies and percentages, while continuous variables are presented as mean ± standard deviation. Chi-square test, Fisher’s exact test two-tailed and *t*-test were used for statistical analysis, as indicated. Any *p*-values lower than 0.05 were considered statistically significant. Sample size calculation was performed to detect a difference of 0.25 logMAR between the mean initial and final BCVA in the study group, at a significance level of 5% and a power of 80%, assuming a standard deviation of 0.5 logMAR. The minimum sample size of the study was 34 cases (*t*-test, matched pairs, two tails, G*Power 3.1.9.7 software).

## 3. Results

Thirty-eight patients (76 eyes), including 27 women (71.05%) and 11 men (28.94%), were included in this study. The female-to-male ratio was 2.45:1. At the end of the follow-up, 10 patients had the complete type of VKH (26.3%), while 28 had the incomplete type (73.7%).

The mean age at uveitis onset was 36.6 ± 12.4 years (median: 37.5 years, range 12–62 years), and the mean follow-up was 120.92 ± 71.78 months (median: 115 months, range: 12–280 months).

The period lasting from the onset of ocular symptoms to the first ophthalmologic evaluation and therapy was 12.97 ± 28.29 days (median 7 days; range 1–180 days), while the period lasting from the onset of ocular symptoms to our first examination was 329.58 ± 662.76 days (median 105 days; range 7–3600 days) (Table 1).

The clinical features seen by us or reported in the patients’ files at disease onset and the complications that occurred during the follow-up of all our patients are reported in Table 1.

At disease onset in primary care centers, choroiditis was diagnosed ophthalmoscopically in all the patients, ten of whom were also investigated with FA and ICG, which confirmed the diagnosis.

The mean visual acuity at the time of the last examination was 0.13 ± 0.4 logMAR, which was statistical increased from the baseline (0. 43 ± 0.33; t = −5.044, *p* < 0.0001), with 96.05% of the eyes experiencing an improvement in visual acuity from the baseline. Twenty-four patients (63.16%) were out of any therapy for a mean period of 56.25 ± 50.57 months (range: 2–212 months), without evidence of recurrences detected ophthalmoscopically or by ICG and/or EDI-OCT.

Table 2 reports the demographics, clinical features, and complications of the patients divided into those treated with oral corticosteroids from uveitis onset (20 patients, group 1) and those who received intravenous corticosteroids at onset (18 patients, group 2).

There were no statistically significant differences between the two groups concerning the mean age at onset of the disease, the period from the onset of symptoms to the patients’ first therapy and to our examination, the mean follow-up duration, or the subsequent use of immunosuppressive drugs (50% in both groups, with a similar period from the onset of symptoms to the start of immunosuppressive therapy and comparable types of drugs and mean durations of treatment) (Table 2). Exudative retinal detachment was more common in the patients treated with intravenous corticosteroids at onset (94.4% vs. 65%, *p* = 0.045), while in this group, there were fewer relapses (49 vs. 157; mean 3.72 ± 3.6 vs. 7.8 ± 6.6, *p* = 0.026), a lower rate of relapse per patient per months of follow-up (0.02 ± 0.03 vs. 0.06 ± 0.02, *p* < 0.0001), and a greater number of patients who did not experience any uveitis relapse after the first course of therapy (44.4% vs. 5%, *p* = 0.007).

We did not observe any differences between the groups concerning the mean amount of prednisone administered, the number of periocular injections and the number of intravitreal dexamethasone implants received, the mean time from the onset of symptoms to the start of immunosuppressive therapy, the type of drug used, or the mean duration of this therapy (Table 2).

Similarly, no differences were found regarding the prevalence of cataracts or IOP increase, while the patients treated with intravenous steroids showed sunset glow fundus (33.3% versus 55%) less frequently and were more likely to be out of therapy (72.2% vs. 55%). The mean period out of therapy without any ocular or systemic manifestation of VKH was 49.85 ± 45.64 months in the patients treated with intravenous corticosteroids and 63.8 ± 59.36 months among the others.

One patient in group 1 had a final visual acuity of light perception due to the onset of secondary glaucoma, unresponsive to medical therapy, for which the patient refused any surgical procedure. After excluding this patient from the analysis and considering each eye separately, we found a significant increase in the mean visual acuity from onset to the last examination in both groups of patients (group 1: right eye from 0.46 ± 0.38 to 0.15 ± 0.24, *p* = 0.0038; left eye from 0.42 ± 0.35 to 0.1 ± 0.29, *p* = 0.0032; group 2: right eye from 0.4 ± 0.24 to 0.03 ± 0.08 *p* < 0.0001, left eye from 0.44 ± 0.34 to 0.06 ± 0.24, *p* < 0.0001).

Further dividing the patients into two groups according to the use of immunosuppressive drugs, 19 subjects (50%) were treated with this regimen during the follow-up. The mean time from the onset of symptoms to the initiation of immunosuppressive therapy was 597 ± 888.19 days (median: 180 days, range: 7–2880 days). The demographics, clinical features, and complications of these two groups of patients (group 3 untreated; group 4 treated with immunosuppressive drugs) are reported in Table 3.

The only significant difference between these groups was the duration of follow-up, which was longer for the patients who did not receive immunosuppressive drugs, and the total dosage of corticosteroid therapy, which was higher for the patients who received immunosuppressive therapy (*p* = 0.021). The clinical features and complications did not differ between the patients treated or untreated with immunosuppressive drugs, while a significant number of the patients untreated with immunosuppressive therapy were out of therapy (*p* = 0.019). Only sunset glow fundus seemed to be more frequent in patients untreated with immunosuppressive therapy (52.6% vs. 36.8%), but this difference did not reach statistical significance (Table 3).

When comparing the patients initially treated with oral or intravenous corticosteroids, who were subsequently treated or untreated with immunosuppressive therapy, none of the main outcomes were significantly different in the comparison between these groups.

Sunset glow fundus developed in 17 out of the 38 patients (44.73%). Peripapillary atrophy was found in all the patients (100%) who were diagnosed with sunset glow fundus. Sunset glow fundus was found in one out of seven patients treated by us within four weeks of the onset of symptoms (14.3%) and in 51.6% of the patients treated elsewhere. Sunset glow fundus developed in 30% of the patients who received intravenous steroids at onset and immunosuppressive drugs thereafter. The mean duration of corticosteroid therapy in the acute phase of the disease differed between the patients with sunset glow fundus (16.6 ± 10.898 months) and those who did not develop it (14.78 ± 6.21), but this difference was not statistically significant. In the patients with sunset glow fundus, cataracts occurred in fourteen cases (82.35%), IOP increased in eight (47.05%), and a neovascular membrane was found in one (5.88%), with the first two findings occurring statistically more frequently than in the patients without sunset glow fundus (*p* < 0.0001 and *p* = 0.037, respectively).

The final visual acuity was significantly higher in the patients without sunset glow fundus (0.03 ± 0.09) compared to those with sunset glow fundus (0.29 ± 0.59) (t = 2.82, *p* = 0.006).

At the last follow-up, 14 patients were still on therapy including eight who were on immunosuppressive drugs only, seven on azathioprine, and one on adalimumab. Additionally, four patients were on low-dose corticosteroids (≤5 mg/day of prednisone) and two were on immunosuppressive therapy and low-dose corticosteroids.

## 4. Discussion

Patients with VKH usually present a good initial response to corticosteroid therapy, delivered either orally or intravenously [1,2,16,17]. In a multicentre study on the use of corticosteroids in acute VKH, no difference could be found in terms of visual recovery and final visual acuity in patients treated with oral corticosteroids when compared with those who received a bolus of intravenous methylprednisolone for three to five days [4].

Nevertheless, no studies are available on the real-life long-term management of these patients in countries where the disease is uncommon, and some patients might access uveitis centers weeks or months after the onset of their disease.

In our patient population, we did not observe any differences in terms of the sex distribution or mean age at onset of the disease compared to other studies from different countries worldwide [1,5,12,18].

Papillitis and choroiditis were reported in all the patients who were subsequently referred to us, while exudative retinal detachment was described in almost 80% of the cases, which is comparable to the results of a study on Chinese patients [19]. VKH is primarily a stromal choroiditis. Therefore, a detailed investigation with ICG and EDI-OCT, which are the most sensitive analyses for the detection and the precise quantification of choroidal involvement, should be performed immediately after the onset of the disease, without waiting for the development of an exudative retinal detachment.

We observed a significant delay between the onset of ocular symptoms and the first ophthalmologic evaluation and therapy (median 7 days) and the timing of referral to tertiary uveitis care center (median 105 days), which might have influenced the route of administration of the corticosteroids and the indication for immunosuppressive therapy. In this scenario, our patients were initially treated with either oral or intravenous corticosteroids, and subjects who were treated intravenously were sent to our center earlier than those who received oral corticosteroids.

We also found that patients presenting with exudative retinal detachment at onset were more frequently treated with intravenous corticosteroids (*p* = 0.045). Therefore, we can conclude that aggressive initial therapy, such as intravenous corticosteroids, is much more closely related to a severe presentation of the disease at onset, and supports the notion that general ophthalmologists should ask for earlier consultations at referral centers.

Nevertheless, the final visual acuity among all the patients with VKH, independent of the initial treatment, was quite good (mean: 0.13 logMAR) and statistically increased from the baseline (*p* < 0.0001).

In a follow-up lasting for a mean period of almost nine years, we were able to demonstrate that intravenous corticosteroid therapy at onset, independent from the administration of additional immunosuppressive therapy, was significantly related to a better course of the disease, showing less (*p* = 0.026) or no recurrence at all after induction therapy (*p* = 0.007), required fewer systemic, periocular injections or intravitreal implants of corticosteroids, and increased the patients’ chances of not requiring further therapy (72% versus 55%).

The VKH-related complications did not differ between the two groups, even though the onset of sunset glow fundus affected about 55% of the cases treated with oral corticosteroids at onset and 33% of those who received intravenous therapy.

Unlike the findings of Read et al. [4], who demonstrated, in a multicentre study, that the route of administration of corticosteroids (oral only or intravenous plus oral) in patients with acute VKH did not affect the final visual prognosis and the rate of complications, our study seems to suggest that the administration of intravenous corticosteroids at disease onset offers beneficial effects. This difference may be due to the shorter follow-up of the multicentre study compared to our investigation (median: 15 months vs. 115 months).

Another important issue in the determination of the most appropriate treatment for VKH patients is the evidence that many patients, despite adequate therapy at VKH onset, might develop chronic recurrent granulomatous inflammation, progressive depigmentation, and chorioretinal atrophy during follow-up, leading to the typical picture of sunset glow fundus, although the disease may appear to be under control [7,8,9,20,21,22].

Several studies have demonstrated that the rate of chronic recurrent disease in all cases of VKH ranges between 17.5 and 79% [7,8,9], despite prompt and adequate treatment during the acute phase. Recently, Abu El Asrar et al. found that the use of mycophenolate after a median time of 9 days and, in all cases, within 30 days of VKH symptoms onset, successfully prevented the onset of both uveitis recurrence and sunset glow fundus [12]. Other authors [9,11,12,13,20,23] have suggested the need to begin immunosuppressive therapy within 2–3 weeks from the onset of symptoms. Conversely, other authors have shown that in some cases, patients can recover very well after a course of corticosteroid therapy alone and, therefore, treating all subjects with VKH might lead to overtreatment, exposing patients to side-effects related to immunosuppressive therapy, which is potentially avoidable [5,17,24]. Additionally, a high-dose course of intravenous corticosteroids, eventually repeated up to three times in case ophthalmoscopic and fluorescein angiography evaluations are not completely negative, was found to lead to good visual recovery, with only 15% of the cases requiring treatment with immunosuppressive drugs [5].

Our study points out that in the real-life management of VKH in countries where this disease is uncommon, it is possible that patients are sent to referral centers late after disease onset, thus reducing their chances of being treated early on with immunosuppressive therapy, which is quite unfamiliar to general ophthalmologists. Therefore, our study cannot add any information about whether immunosuppressive therapy given within a few days of the onset of symptoms, as proposed by some authors [9,11,12,13,19,23], is more effective than treating patients with corticosteroids only, because our patients were treated with immunosuppressive therapy after a median period of six months from the onset of their ocular symptoms. Nevertheless, we checked whether immunosuppressive therapy, independent of the initial method of corticosteroid administration, could influence the clinical features and final visual prognosis in a VKH population. For this situation, we did not record any statistical difference in terms of the mean number of relapses, the rate of relapses per patient per month of follow-up, the final visual acuity, and the frequency of complications, although sunset glow fundus was found more frequently in the patients left untreated by immunosuppressive drugs (53% vs. 37%).

The rate of complications found in our series was higher than those reported by other authors. Cataract and IOP increases were diagnosed in 36.8% and 31.6%, respectively, of our patients treated with corticosteroids and immunosuppressive drugs, in contrast to rates such as the 6.6% and 2.6%, respectively, found in Saudi Arabia [12] and 18% and 4.5%, respectively, in Japan [5]. Our results were more similar to those detected in China (28.85% and 25.95%, respectively) [19]. The longer follow-up of our patients might itself explain these differences, although the role of corticosteroid therapy cannot be disregarded, because the cumulative dose of corticosteroids administered to the patients was not reported in the other series.

Sunset glow fundus is a typical late complication of VKH. It has been associated with a worse visual prognosis, higher rates of complications [6], and mean retinal sensitivity [25], although one study reported an incidence of 49.5% of sunset glow fundus with a final visual acuity greater than 1.0 in 94.4% of cases after five years of follow-up [5]. Recently, Lin Oo et al. reported a rate of development of sunset glow fundus of 57.7% in their patients, although all of these patients were treated with corticosteroids and immunosuppressive therapy within 3 months of symptom onset [23]. We can confirm that sunset glow fundus might occur globally in 44% of VKH cases, but that this figure reduces to 14% if patients are treated at a referral center for uveitis. In our experience, sunset glow fundus is statistically associated with a worse final visual acuity (*p* = 0.006) and higher rates of cataract and IOP increase (*p* < 0.001 and *p* = 0.037, respectively). Delayed immunosuppressive therapy was proven to reduce the occurrence of sunset glow fundus in our patients (from 53% to 37%), thus indirectly improving the final visual prognosis and suggesting a wider use of immunosuppressive therapy.

A limitation of our study is the lack of a precise assessment of the choroidal involvement at the disease’s onset by ICG and EDI-OCT, because all the patients were previously treated elsewhere. This assessment, if performed initially and repeated during the follow-up, can be used to define the exact correlation between the extension of choroidal involvement at onset and the potential subsequent development of sunset glow fundus [26,27].

## 5. Conclusions

Our study on the real-life management of VKH in Italy demonstrated that it is not uncommon for patients to be examined later in the course of their disease. In such situations, intravenous corticosteroid therapy administered at symptom onset may significantly reduce the onset of uveitis recurrence during a long-term mean follow-up, theoretically lessening the probability of losing visual acuity. Moreover, the use of immunosuppressive therapy, even if it is not given at the onset of VKH but, instead, during the course of the disease, might decrease the occurrence of sunset glow fundus, thus lowering the rate of complications and improving the visual prognosis. Patients in whom VKH is suspected or diagnosed should be sent to uveitis centers for proper and prompt treatment, including, if needed, immunosuppressive therapy, periocular injections, or intravitreal implants of corticosteroids.

## Figures and Tables

**Table 1:** Demographics and clinical features of 38 patients with Vogt–Koyanagi–Harada disease.

Number of patients	38
Age at onset (mean)	36.6 ± 12.4 years
Female	27 (71.05%)
Follow-up (months)	
mean	120.92 ± 71.78
median	115
Time period from onset of ocular symptoms to first examination and therapy (days)	
mean	12.97 ± 28.29
median	7
Time period from onset of ocular symptoms to our examination (days)	
mean	329.58 ± 662.76
median	105
Immunosuppressive therapy during follow-up	19 (50%)
Clinical findings at uveitis onset:	
- Exudative retinal detachment	30 (78.9%)
- Papillitis	38 (100%)
- Choroiditis	38 (100%)
- Anterior uveitis	11 (29.9%)
Mean initial visual acuity Mean final visual acuity * *p* < 0.0001	0.43 ± 0.33 * 0.13 ± 0.4 *
Complications at last examination:	
- Cataract	17 (44.73%)
- Intraocular pressure increase	11 (28.94%)
- Neovascular membrane	1 (2.6%)
- Sunset glow fundus	17 (44.73%)
N° (%) of patients out of therapy	24 (63.16%)
mean time (months)	56.25 ± 50.57

**Table 2:** Demographics and clinical features of patients with Vogt–Koyanagi–Harada disease according to the route of administration of corticosteroids at onset: group 1 oral and group 2 intravenous corticosteroids.

	Group 1 Oral Steroids at Onset	Group 2 Intravenous Steroids at Onset	Chi-Square Fisher’s Exact Test or *t*-Test	*p*
Number of patients	20	18		
Age at onset	36.05 ± 12.82	37.83 ± 12	−0.44	0.662
Female	15 (75%)	12 (66.6%)	0.043	0.836
Follow-up (months)	117.8 ± 70.49	96.72 ± 61.12	0.98	0.334
Time period from ocular symptoms onset to therapy (days)	16.15 ± 38.83	9.44 ± 5.64	0.725	0.473
Time period from ocular symptoms onset and our examination (days)	455.9 ± 783.73	189.22 ± 479.5	1.248	0.22
Clinical findings at uveitis onset				
- Exudative retinal detachment	13/20 (65%)	17/18 (94.4%)	-	0.045
- Papillitis	20 (100%)	18 (100%)	-	1
- Choroiditis	20 (100%)	18 (100%)	-	1
- Anterior uveitis	6 (30%)	5 (27.7%)	-	1
Mean amount of corticosteroid therapy (mg of prednisone)	23,679.28 ± 20,023.22 median: 15.163	18,509.39 ± 17,225.16 median: 12.318	0.848	0.402
Total number of periocular injections (triamcinolone/methylprednisolone 40 mg)	53 in 10 patients (50%)	30 in 6 patients (33.3%)	0.504	0.478
Total number of dexamethasone intravitreal implants (700 mg)	7 in 4 patients (20%)	0	-	0.11
Immunosuppressive therapy	10/20 (50%)	9/18 (50%)	0.106	0.745
▪ Mean time from symptom onset to therapy (days)	783 ± 911.37	410.77 ± 761.3	−0.90	0.188
▪ First immunosuppressive drug				
- azathioprine	6	9	-	0.0867
- methotrexate	2	0	-	0.4737
- cyclosporine	2	0	-	0.4737
▪ Multiple immunosuppressive drugs during follow-up				
- cyclosporine, methotrexate, azathioprine, adalimumab	1	0	-	1
- cyclosporine, azatioprine	1	0	-	1
- azathioprine + adalimumab	0	2	-	1
▪ Mean duration of therapy (months)	62.30 ± 71.85	51.44 ± 26.1	−0.428	0.674
Total number of relapses	157	49		
Patients without relapses	1/20 (5%)	8/18 (44.4%)	-	0.007
Mean number of relapses	7.8 ± 6.6	3.72 ± 3.69	2.315	0.026
Relapse/patient/month of follow-up	0.06 ± 0.03	0.02 ± 0.03	4.104	<0.001
Right eye: initial visual acuity	0.45 ± 0.38	0.4 ± 0.24	0.479	0.635
Right eye: final visual acuity	0.29 ±0.68	0.03 ± 0.08	1.61	0.116
Left eye: initial visual acuity	0.42 ± 0.35	0.44 ± 0.34	−0.178	0.86
Left eye: final visual acuity	0.1 ± 0.29	0.06 ± 0.24	0.46	0.648
Complications				
- Cataracts	9/20 (45%)	8/17 (47.06%)	0.042	0.837
- Intraocular pressure increase	5/20 (25%)	6/18 (33.3%)	0.043	0.836
mean peak value	33.4 ± 15.2 mmHg	30.5 ± 6.1 mmHg	0.433	0.675
	range 24–60 mmHg	range 24–40 mmHg		
- Neovascular membrane	1/20 (5%)	0/18 (0%)	-	1
Sunset glow fundus	11/20 (55%)	6/18 (33.3%)	1.029	0.31
N° (%) of patients out of therapy	11/20 (55%)	13/18 (72.2%)	0.581	0.446
mean time (months)	63.82 ± 59.36	49.85 ± 45.64	0.806	0.425

**Table 3:** Demographics and clinical features of patients with Vogt–Koyanagi–Harada disease, according to the administration of immunosuppressive drugs during follow-up.

	Group 3 NO Immunosuppressive Therapy	Group 4 YES Immunosuppressive Therapy	Chi-Square Fisher’s Exact Test or *t*-Test	*p*
Number of patients	19	19		
Age at onset	35.63 ± 14.34	37.63 ± 10.48	−0.491	0.627
Female	11 (57.9%)	16 (84.2%)	2.047	0.152
Follow-up (months)	151.05 ± 63.49	97.32 ± 66.33	2.551	0.015
Time period from the onset of ocular symptoms to first therapy	16.58 ± 39.81	9.37 ± 5.79	0.781	0.44
Time period from the onset of ocular symptoms to our examination (days)	393.16 ± 820.93	266 ± 469.53	0.586	0.561
Mean amount of corticosteroid therapy (mg of prednisone)	14,034.4 ± 7558.11	27,420 ± 22,316	−2.415	0.021
Total number of periocular injection (triamcinolone/methylprednisolone 40 mg)	27 in 6 patients 31.57%	52 in 10 patients 52.63%	0.504	0.478
Total number of dexamethasone intravitreal implants (700 mg)	0	7 in 4 patients 21.05%	-	0.105
Clinical findings at first examination				
- Exudative retinal detachment	11 (57.89%)	14 (73.68%)	-	0.495
- Papillitis	19 (100%)	19 (100%)	-	1
- Choroiditis	19 (100%)	19 (100%)	-	1
- Anterior uveitis	7 (36.8%)	4 (21%)	-	0.476
Total number of relapses	109	86		
Patients without relapses	5 (26.31%)	4 (21.05%)	-	1
Mean number of relapses	5.74 ± 6.45	4.53 ± 5.08	0.642	0.525
Relapse/patient/month of follow-up	0.04 ± 0.04	0.03 ± 0.03	0.872	0.389
Right eye: initial visual acuity	0.38 ± 0.3	0.48 ± 0.32	−0.994	0.327
Right eye: final visual acuity	0.25 ± 0.69	0.09 ± 0.19	0.974	0.336
Left eye: initial visual acuity	0.32 ± 0.3	0.55 ± 0.35	−2.175	0.036
Left eye: final visual acuity	0.08 ± 0.23	0.09 ± 0.3	−0.155	0.909
Complications				
- Cataract	7/18 (38.9%)	7/19 (36.84%)	0.044	0.833
- Intraocular pressure increase	5/19 (26.31%)	6/19 (31.57%)	0	1
mean peak value	37.4 ± 13.85 mmHg	28.83 ± 5.88 mmHg	1.385	0.199
	range 24–60 mmHg	range 24–38 mmHg		
- Neovascular membrane	1/19 (5.26%)	0/19 (0%)	0	1
Sunset glow fundus	10/19 (52.63%)	7/19 (36.84%)	0.426	0.514
N° (%) of patients out of therapy	16/19 (84.2%)	8/19 (42.1%)	5.542	0.019
mean time (months)	69.25 ± 61.49	42.75 ± 24.12	1.749	0.089

## Data Availability

Not applicable.
